# RNAthermsw: Direct Temperature Simulations for Predicting the Location of RNA Thermometers

**DOI:** 10.1371/journal.pone.0094340

**Published:** 2014-04-09

**Authors:** Alexander Churkin, Assaf Avihoo, Michal Shapira, Danny Barash

**Affiliations:** 1 Department of Computer Science, Ben-Gurion University, Beer-Sheva, Israel; 2 Microsoft Research Israel, Herzliya, Israel; 3 Department of Life Sciences, Ben-Gurion University, Beer-Sheva, Israel; University of South Florida College of Medicine, United States of America

## Abstract

The mechanism of RNA thermometers is a subject of growing interest. Also known as RNA thermosensors, these temperature-sensitive segments of the mRNA regulate gene expression by changing their secondary structure in response to temperature fluctuations. The detection of RNA thermometers in various genes of interest is valuable as it could lead to the discovery of new thermometers participating in fundamental processes such as preferential translation during heat-shock. RNAthermsw is a user-friendly webserver for predicting the location of RNA thermometers using direct temperature simulations. It operates by analyzing dotted figures generated as a result of a moving window that performs successive energy minimization folding predictions. Inputs include the RNA sequence, window size, and desired temperature change. RNAthermsw can be freely accessed at http://www.cs.bgu.ac.il/~rnathemsw/RNAthemsw/ (with the slash sign at the end). The website contains a help page with explanations regarding the exact usage.

## Introduction

RNA thermometers are temperature-sensitive RNAs that regulate gene expression and have recently attracted growing attention [13]. They are also known as RNA thermosensors and in general, they alter their secondary structure in response to temperature fluctuations. Together with riboswitches [14], [20], these peculiar RNA switches are used as examples in support of the RNA world hypothesis. Specific examples of RNA thermometers include FourU [23], Hsp90 *cis*-regulatory element [1], ROSE element [19] and Hsp17 thermometer [12]. RNA thermometers typically regulate genes required during either a heat shock or a cold shock response, but have implicated in other regulatory roles such as in pathogenicity and starvation [18]. Their conformational change and 3D structure have also been investigated [4], [5]. The simplest RNA thermometers have a single-hairpin structure but some are significantly more complex, as in the CspA mRNA [8]. Bioinformatics search methods have been used to identify novel candidate RNA thermometers [22]. Structure-based methods are needed for RNA thermometers because phylogenetic comparisons showed that the secondary structure of the temperature-sensitive RNA element is much more conserved than the nucleic acid sequence [22]. Thus, bioinformatic search methods for RNA thermometers should be focused on structural properties rather than on the sequence. The approach taken in [22] was to establish a searchable database called RNA-SURIBA (Structures of Untranslated Regions In BActeria). The search in this database was completely independent of the primary sequence. Storage of structures in RNA-SURIBA involved RNA secondary structure prediction by energy minimization using mfold [27] and dot bracket annotation together with relevant characteristics such as number of hairpins and bulges. Database search was performed with regular expression for the dot bracket annotation combined with structural features. However, the approach is limited to searching a database composed of known RNA thermometers with their corresponding structures. Here, relying on energy minimization as in [22] but also including prediction of temperature changes, a simulation of heat-shock and cold-shock is performed. Energy minimization studies with temperature changes using the Vienna RNA package [9] for exploring synthetic and natural RNA thermometers have been performed before [2], [21], but not for attempting to identify locations of RNA thermometers. The motivation of RNAthermsw is to identify locations of RNA thermometers by the direct simulation of temperatures. It relies on Vienna’s RNAfold [10] with energy parameters taken from the most recent Turner model [16]. The initial program was reported in [3] and is conceptually based on a sliding window with local folding predictions performed inside the moving window, as in [11].

At the time of completion of the RNAthermsw webserver, we became aware of the RNAtips webserver [6] that was recently published. These two applications were developed independently, with RNAthermsw relying on method details and results described in [3]. The differences between the two are referred to in the Discussion.

## Methods

### Preliminaries

The era of high throughput sequencing provides new opportunities for the identification of RNA segments that regulate gene expression. Innovative experimental protocols (e.g., [17]) are becoming available to study RNA structure and interactions in vitro in a variety of conditions. Already, thousands of putative RNA thermometers were identified by probing yeast RNA structures at different temperatures [25]. Genome-wide profiling of RNA secondary structure are showing features that may allow RNAs associated with stress responses to undergo conformational changes in response to environmental conditions [7]. The possibility to map and analyze RNA structure transcriptome-wide across a range of temperatures, finding key roles for temperature-sensitive RNA motifs, motivates the inspection of particular genes at higher resolution to identify new RNA thermometers in the 5′ UTR of eukaryotes where little is known about them in comparison to bacteria. RNAthermsw attempts to determine if certain RNA molecules alter their structure in silico. Using RNAthermsw, we seek to identify relatively short (50–80 nt long), prone to change segments of an RNA sequence. Initially, the input is a long sequence, often a 5′ UTR, that is suspected to manifest the capacity to alternate between two energetically stable structures. The whole sequence is scanned using the window technique outlined below, intending to reveal at least a small number of conformational rearranging segment candidates in response to temperature fluctuations that can be further tested experimentally.

### Sliding Window and Window Size Variation

It is well known that a structure prediction program based solely on conformational rules and thermodynamics will become increasingly inaccurate for long RNA sequences that are several hundred nucleotides long [15], [26]. Because RNA folding predictions by energy minimization cannot accurately predict the folding of complete 5′ UTRs that are rather long, the sequences have to be chopped into smaller segments (windows) for which accurate folding predictions are possible. To preserve the global fold of the larger RNA sequence when dividing it into smaller segments, the window slides over the entire sequence one nucleotide at a time; each single nucleotide shift reveals a new, small segment whose fold can be separately tested. Because the complete sequence is cut into the window-defined segments, there is no ideal size for the segments. The original sequence may display folding behavior different from that of a particular segment, and by varying the window’s size more information can be derived from the same sequence via the analysis of its parts. Thus, a range of window sizes from smallest to largest is considered.

### Forming the Output and the Prediction

**Figure 1:**
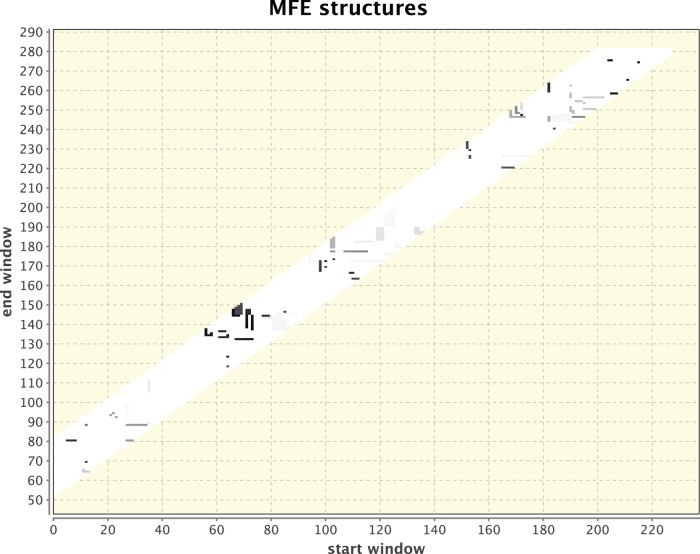
Simulated heat-shock response for *homo sapiens* 70 kDa HSPA4. Parameter values are: windows: 55–80, temperatures: 37°C and 40°C. Sequence size is 281 nt. In inspecting the graphical plot, there is no special clustering of dots to indicate a signal for a potential RNA thermometer. The structural changes are sporadically distributed over the entire RNA sequence.

**Figure 2:**
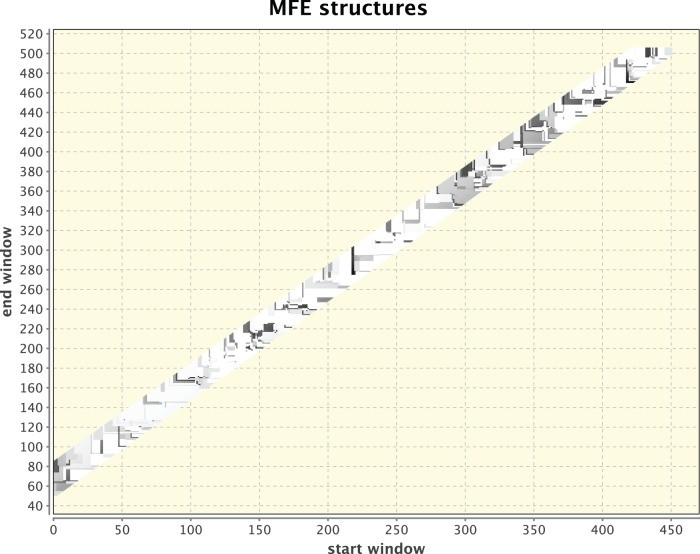
Simulated heat-shock response for *drosophila melanogaster* HSP70 Aa. Parameter values are: windows: 55–80, temperatures: 25°C and 40°C. Sequence size is 503 nt. In inspecting the graphical plot, a significant cluster of RNA structural activity occurs at segments around 300 and 350, indicating a signal for a potential RNA thermometer.

RNAthermsw uses a sliding window approach to traverse a region of interest in the genome that may contain an RNA thermometer. Each time, it performs two folding predictions by energy minimization inside the given window. The first is with a default temperature and the second is with a specified change in temperature that is intended to approximately simulate a heat-shock or a cold-shock effect. The change in temperature is determined by the user according to the biological context and the biochemical experiment that is performed, noting that it is easier to perform computational simulations to explore a variety of temperature ranges than to conduct wet lab experiments. The difference between the two secondary structures predicted is then calculated by the base pair distance, after which a dot is displayed with intensity that is proportional to the distance. If there is no change in secondary structure then the dot is white (invisible), while for the largest distance the dot is black. If the dots are plotted on a graph in which the x-axis corresponds to the left-most position of the window inside the region of interest and the y-axis corresponds to the right-most position, as in Figures 1 & 2, a plot is obtained whereby a pronounced cluster of visible dots indicates a potential RNA thermometer. Thus, each segment defined by the window is assessed as to whether and to what extent it undergoes a structural change. The beginnings (x) and the ends (y) of all the segments exhibiting changes in structure form dots on the xy plane, thereby forming the initial, predictive output: clusters on the RNA sequence containing multiple segments susceptible to structural changes will show up on the two dimensional plane as relatively dense and dark concentrations of dots. This initial output is used to identify areas of the RNA sequence most likely to cause structural change in the RNA molecule. Once the dot plot is output, the user needs to qualitatively evaluate the dot plot in order to decide which regions to examine more closely. The relevant segments in highly dense regions hold the most promise for RNA structural activity.

Additional information and a detailed example of software usage are available at http://www.cs.bgu.ac.il/~rnathemsw/RNAthemsw/help.html. The server itself is accessible at http://www.cs.bgu.ac.il/~rnathemsw/RNAthemsw/ (note the slash sign at the end). The output of each run is a dotted figure in which the formation of noticeable clusters, if available, are looked for. The most interesting locations of the clusters can be used for experimental validation.

## Results and Discussion

**Figure 3:**
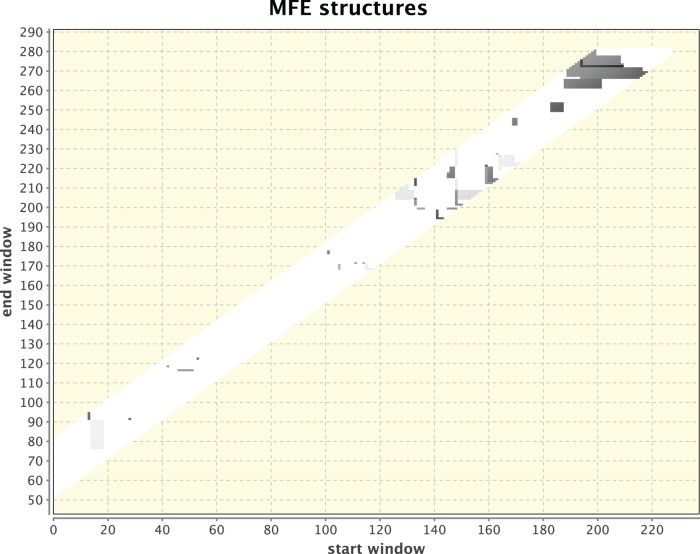
Simulated heat-shock response for a sequence containing the *E.coli ibpA* RNA thermometer. A clustering of dots indicating an RNA thermometer is observed.

**Figure 4:**
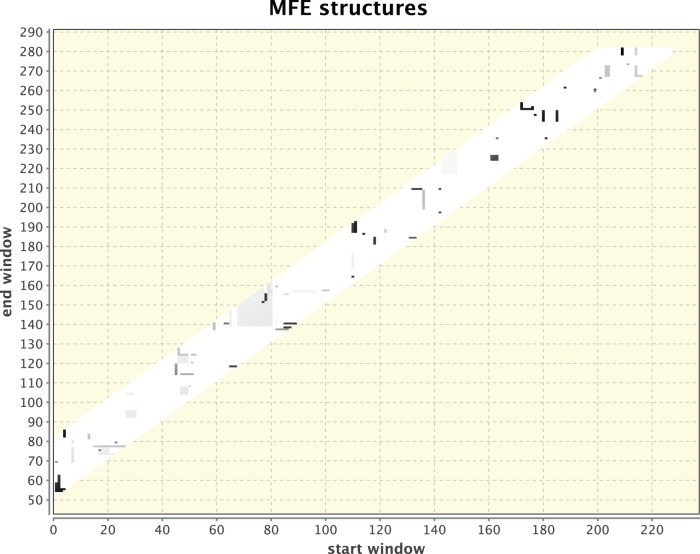
Simulated heat-shock response for a control sequence in the vicinity of the *E.coli ibpA* RNA thermometer, but not containing the thermometer itself. No clustering of dots that may indicate a potential RNA thermometer is observed.

For method illustration, Figures 1 & 2 respectively depict two independent runs of 5′ UTRs that were isolated from two different organisms: Drosophila and humans. Each was kept at its body temperature and a different, elevated temperature was chosen to elicit the heat-shock response. While the dots in Figure 1 are dispersed without any singular cluster, the clustering of dots around positions 300 and 350 in Figure 2 provides a signal for a potential RNA thermometer that should be examined further. Other smaller clusters can also be observed and in principle should be checked, but the most pronounced cluster holds the most promise to relate to an RNA thermometer because of the manner in which the dotted figures are generated. It should be noted that as can be observed in the next example that serves for validation of the method, which is the detection of a known RNA thermometer in Figures 3 & 4, the signal is not a consequence of having a longer sequence as input or simulating a larger difference in temperature because of a lower body temperature in Drosophila than in humans. A check (not shown) was performed when comparing a part of the *drosophila melanogaster* HSP70 gene that is equal in its sequence length to that of the *homo sapiens* HSP70 gene and raising the temperature difference in humans to be the same as in Drosophila. A signal was still found for a potential RNA thermometer appearing in the HSP70 of Drosophila that does not appear in the HSP70 of humans.

The reason for examining HSP (Heat-Shock Protein) genes in various eukaryotic organisms has been outlined in the previous section in relation to contemporary high-throughput sequencing attempts to analyze RNA secondary structures genome-wide [7], [17], [25]. High-throughput sequencing reveals, for example, that mRNAs associated with stress responses tend to be more single-stranded than others, with longer maximal loop length and higher free energy per nucleotide, features which may allow these RNAs to undergo conformational changes in response to heat-shock. The HSP70 gene in Drosophila and in humans shown in Figures 1 & 2 is an illustration of how RNAthermsw can be used to look for RNA thermometers in various HSP genes at various organisms by a direct simulation of temperature fluctuations. Starting with the desired RNA sequence, we used 5′ UTR of heat shock proteins (HSPs) varying in length from 250 nt to 500 nt, too large for a reliable folding prediction of the entire sequence. The 5′ UTRs were isolated from different organisms to determine if correlation exists in the folding behavior of the RNA of different organisms. Each organism was kept at its body temperature, and a different, elevated temperature was chosen to elicit the heat shock response. At present, there are still no structure mapping datasets for humans and Drosophila that can be compared to our predictions. Nevertheless, the discovery of heat-shock proteins was made in Drosophila and our interest in these organisms motivated our selection for illustration. In the future, we anticipate that structure mapping datasets will be available and we can also test our predictions using single-gene experiments.

Another illustration is available in Figures 3 & 4 in which the *E.coli ibpA* known RNA thermometer [24] was used to validate the method and to demonstrate its potential. The thermometer itself as appears in [24] is 102 nt long. Around 40 nt were taken upstream and downstream the known RNA thermometer to construct a sequence of 281 nt (as in the case of Figure 1, without loss of generality) that contains the thermometer. A control sequence that contains no RNA thermometer was taken from the vicinity, several hundred nucleotides away, with the same length of 281 nt. The parameter values used for these two sequences were the same (windows: 55–80, temperatures: 35°C and 37°C). The cluster in Figure 3 that is missing in Figure 4 is clearly observed, validating that the sequence in Figure 3 contains a known RNA thermometer. RNAthermsw supports a different plot for centroid structures in addition to the plot for MFE structures. The several window sizes that are chosen are integrated into the dotted plot output, which can be observed by the fact that the plot contains a vertical band and is not strictly linear. A minimum window size that is less than 50 or a maximum window size that is more than 150 could make energy minimization predictions less reliable, since the accuracy of predictions may fall as the sequence size increases [15]. For this reason and because of efficiency considerations with respect to computation time, we have chosen to work with a window range of 50–80, although for some RNAs such as viruses that are well predicted by energy minimization this range can increase. Another limitation worthwhile mentioning is that long range interactions could exist, which fall outside of the chosen window sizes.

As was mentioned in the Introduction, at the time of completion of the RNAthermsw webserver we became aware of the RNAtips webserver [6] that was recently published. The two applications were developed independently, with RNAthermsw relying on method details and results described in [3]. While RNAtips is based on statistical evaluation for clusters identification after simulating each temperature within a given range, as explained below, RNAthermsw uses multiple windows with a window-size range selected by the user, and the dotted plots in the output provide a direct intuitive way for the user’s interpretation. We have experimented with known RNA thermometers and the perspective gained, also with respect to [22], is important.

### Comparison of RNAthermsw and RNAtips with RNA-SURIBA

The methodology of RNAtips is fully described in [6] and is related to RNAthermsw by the use of RNAfold [9] with varied temperatures in its core. Briefly stated, in the first step, probabilities of nucleotides to be base-paired are assessed at each temperature within the given range by using RNAfold. In the second step, for each nucleotide, RNAtips computes the difference in probability for it to be in a paired state at the lower temperature and at the higher one. These differences are calculated for the entire temperature range and then combined into one data set. In the third step, RNAtips identifies the most temperature-sensitive positions. This is performed by selecting those values (and their corresponding nucleotides) from the data set generated in Step 2, which are distant from the mean by more than three standard deviations. RNAtips then considers these positions to be the most temperature-sensitive, and they are then mapped on the original sequence. Clusters of significantly changing positions are then identified by applying the density-based spatial clustering of applications with noise (DBSCAN) algorithm to the locations of such positions.

It should be stressed again that both RNAtips and RNAthermsw are using RNAfold [9] with varied temperatures in their core. Therefore, one should be aware of the limitation that because of the energy parameters employed, parameters at temperatures other than 37°C can be extrapolated but for temperatures far away from 37°C the results will be increasingly unreliable. However, to justify this type of predictions, the parameters are in constant improvement [15], [16] and they could be enough to show a trend that provides a hint for a potential RNA thermometer. RNA-SURIBA does not suffer from this limitation but it can only identify previously known RNA thermometers, without simulating a temperature change. In general, all three methods employ energy parameters [26], [9], [15], [16] and rely on energy-minimization predictions.

Table 1 provides a high-level comparison between the three methods.

**Table 1:** Comparison between RNAthermsw, RNAtips, and RNA-SURIBA.

Method	Use energy-minimizationpredictions?	Use varyingtemperaturesin predictions?	Based onknown RNAthermometers?	Contain statisticalevaluation?	Use multiplewindow sizes?
RNAthermsw	Yes	Yes	No	No	Yes
RNAtips	Yes	Yes	No	Yes	No
RNASURIBA	Yes	No	Yes	N/A	N/A

When efficiency is considered, it is obvious that RNA-SURIBA is more efficient than RNAthermsw and RNAtips because there is no sliding window in RNA-SURIBA and the bottleneck of computation time, which is the energy-minimization prediction, is performed for only the standard temperature of 37°C. This restricts RNA-SURIBA to identification of previously knows RNA thermometers, without any attempt to simulate heat-shock or cold-shock. The timing of RNAthermsw and RNAtip is comparable since both can be reduced to only a single window and only two temperatures, calling RNAfold the same number of times. However, an advantage of RNAthermsw is the capability of multiple window sizes, whereas the advantage of RNAtips is the capability of simulating each temperature within a given range and performing a statistical evaluation on this data that consists of several temperatures. Each one of these extensions will lead to more calls of RNAfold and will sacrifice efficiency with the purpose of gaining better accuracy.

In terms of accuracy, RNA-SURIBA is expected to be more accurate in identifying previously known RNA thermometers because it is restricted to only this type of search and does not attempt to simulate heat-shock or cold-shock. All methods rely on energy minimization predictions. However, RNAthermsw and RNAtips are relying on energy minimization predictions beyond the standard temperature of 37°C. In this way, their accuracy is comparable, recalling the aforementioned advantage in each (multiple window sizes for RNAthermsw and simulating each temperature within a given range for RNAtips) that sacrifices efficiency with the purpose of gaining better accuracy.

Finally, although no running example on a previously known RNA thermometer using RNAtips is available in [6], we have inserted our example of the sequence containing *E.coli ibpA* from [24] that we tested in Figures 3 & 4 (also available as an example in our online help page) to RNAtips. The RNAtips webserver was able to predict a cluster in position 39–51, although the *E.coli ibpA* from [24] only starts at position 93 and the segment in the RNA thermometer that changes its structure begins at position 164. Admittedly, RNAthermsw may also have other drawbacks besides its successful prediction in this case, and future versions of RNAtips may improve accordingly. The availability of both methods that were developed independently could benefit each other.

## Conclusion

**Figure 5:**
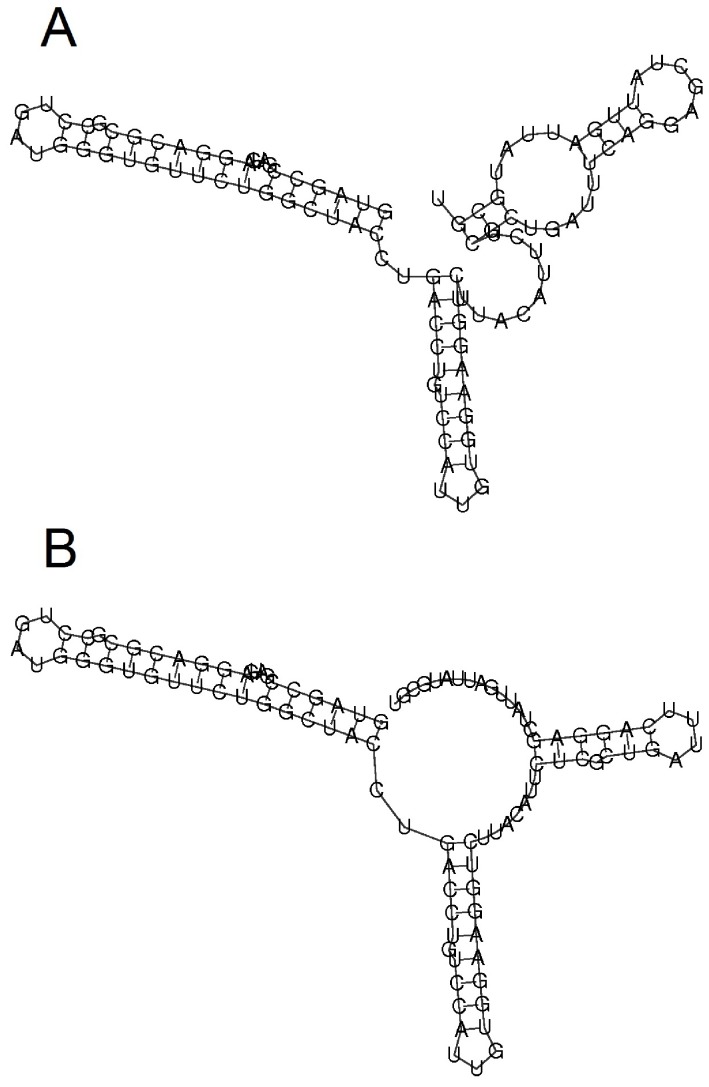
Predicted secondary structures of the *E. coli ibpA* thermometer [24] at 30°C (left) and 45°C (right). Energy minimization predictions were performed using Vienna’s RNAfold webserver, with the most recent energy parameters (Turner model, 2004).

The basic implementation of RNAthermsw does not rely on known RNA thermometers as potential guides to search for new ones. In fact, aside of the *E.coli ibpA* in [24], several known RNA thermometers including the FourU in *Salmonella* [23] and the Repression Of Heat Shock gene Expression (ROSE) element in *Bradyrhizobium japonicum* [18] do not exhibit a change in their predicted structure as a consequence of temperature fluctuations. Therefore the RNA-SURIBA approach [22] should still be used as the method of choice when searching for regions that are similar in their secondary structure to previously known RNA thermometers. The way to check whether a known RNA thermometer would exhibit a change in its predicted structure as a response to temperature fluctuations is to experiment with mfold [27] or RNAfold from the Vienna RNA secondary structure server [10], as shown in Figure 5 for the *E.coli ibpA* known RNA thermometer. Without this check, RNAthermsw can be used to search for completely new RNA thermometers that have not been discovered before, but the identification of previously known RNA thermometers in new locations should proceed with caution.

As more RNA thermometers are discovered, it could be valuable to insert an option into RNAthermsw to make use of known structural information, similar to the approach taken in [22]. This may also confine the search into examining only certain structures but if provided as an optional mode, the detection can be performed in parallel using two separate modes. In addition, a quantitative measurement can be inserted that decides if a structural change is statistically significant, which could be done in the future when more biological experiments that correspond to various computational results (e.g., of 5′ UTR isolated from different organisms) are available. Another possible extension concerns how the mRNA is modelled during translation, as was also suggested in [21]. In the future, incorporating more information to the model such as the effect of ribosomes on the mRNA secondary structure during translation may offer an improved guide along with simulating temperature changes.

## Supporting Information

File S1 **Supplementary information.** The file contains the relevant chapter (Chapter 4) from reference [3].
(PDF)
