# Supplementary material for: RNAthermsw: Direct Temperature Simulations for Predicting the Location of RNA Thermometers
Source: PLoS One. 2014 Apr 9;9(4):e94340. doi: 10.1371/journal.pone.0094340 (PMC3981793; doi:10.1371/journal.pone.0094340)
Supplement: File S1 — Supplementary information. The file contains the relevant chapter (Chapter 4) from reference [3]. (PDF) [file pone.0094340.s001.pdf]

4 Identifying RNA segments that exhibit structural change

When faced with an RNA molecule, the interesting question for the biologist is whether the molecule undergoes structural changes. Often the RNA sequences that biologists work on, for instance the 5'UTR, are too long (over 100 nt) to make accurate predictions about their folding characteristics. One solution is to divide the sequence into "windows" comprising manageable sized segments of less than one hundred base pairs. The segments are identified as those within the window as it slides over the RNA sequence, advancing one nucleotide at a time to define new segments. Each such segment is folded separately (since this was done prior to Vienna 1.6), and the folding data from all the segments are assembled to produce an overall "picture" of the entire RNA sequence. This technique can provide a good indication as to whether the RNA sequence changes its secondary structure. We describe a computational method based on the windows technique for elucidating the folding behavior of RNA's secondary structure.

The existence of two distinct RNA structures with similar free energy values allows for the metamorphosis of RNA molecules in response to some trigger, such as a temperature change. Switching between structures requires the crossing of an energy barrier, and computer simulations can elucidate the characteristics of the energy barrier and of the corresponding RNA structures by predicting the molecule's secondary structure. The segments of the RNA sequence defined by the windows are folded individually and assessed for their robustness in terms of temperature change. If a substantial change occurs in

a particular segment between its native state and how it folds in response to an alternate temperature , then that window is marked as vulnerable. The occurrence of a cluster of vulnerable windows indicates an RNA zone with the potential to function as an RNA switch; in other words, the temperature change trigger is sufficient to alter the molecule's secondary structure between two energetically close stable states.

4.1 Motivation and goals

Biologists often encounter genes that may be regulated by structural changes of RNA, and the only way to verify whether RNA is involved is to identify the specific RNA molecule that undergoes structural changes. The search for such an RNA, however, is an extremely tedious and frustrating endeavor. Our system can assist biologists and save them time in identifying the elusive RNA switches by determining if certain RNA molecules alter their structures in silico.

Using our system, we seek to identify relatively short (60-80 nt long), prone to change segments of an RNA sequence. Initially, the input is a long sequence, often a 5'UTR, that is suspected to manifest the capacity to alternate between two energetically stable structures. The whole sequence is scanned using the window technique outlined above, often revealing at least a small number of switch candidates that can be tested experimentally to determine whether any of them fulfill the switch requirements.

4.2 Description of the problem

Define $A = \{a|a \in (C, G, A, U)\}$

Let A^+ be an instance of the problem, which is an RNA sequence. Only A^+ is used to determine if there is a structural switch.

Many genes have a regulatory site at the gene's 5'UTR, and usually that site is over 200 nt long. We wish to determine whether that 5'UTR will undergo a structural change because of a change in temperature as well as the specific temperature that triggers the transformation.

4.3 Sliding window and window size variation together

Because Vienna and Mfold predictions cannot accurately measure 5'UTRs that are over 200 nt long, the sequences had to be chopped into smaller segments (windows) for which accurate folding predictions are possible. To preserve the globality of the larger RNA sequence when dividing it into smaller segments, the window slides over the entire sequence one nucleotide at a time; each single nucleotide advance reveals a new, small segment whose folding ability can be separately tested. Because the complete sequence is cut into the window-defined segments, there is no ideal size for the segments. The original sequence may display folding behavior different from that of a particular segment, and by varying the window's size more information can be derived from the same sequence via the analysis of its parts.

4.4 Forming the output and the prediction

Each segment defined by the window is assessed as to whether and to what extent it undergoes structural change. The beginnings (x) and the ends (y) of all the segments exhibiting changes in structure form dots on the xy axis, thus forming the initial, predictive output: clusters on the RNA sequence containing multiple segments susceptible to structural changes will show up on the two dimensional plane as relatively dense concentrations of dots. This initial output is used to identify areas of the RNA sequence most likely to cause the structural change in the RNA molecule. These segments hold the most promise for RNA switch activity.

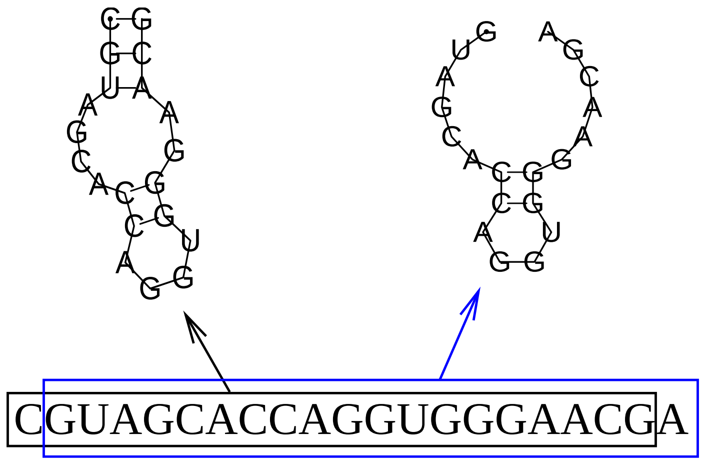

Figure 14: Sliding window.

4.5 Results

Starting with the desired RNA sequence, we used 5'UTR of heat shock proteins (HSPs) varying in length from 250 nt to 500 nt, too large for a direct and reliable folding prediction. The 5'UTRs were isolated from different or-

ganisms to determine if a correlation exists in the folding behavior of the RNA of different organisms. Each organism was kept at its body temperature, and a different, elevated temperature was chosen to elicit the heat shock response.

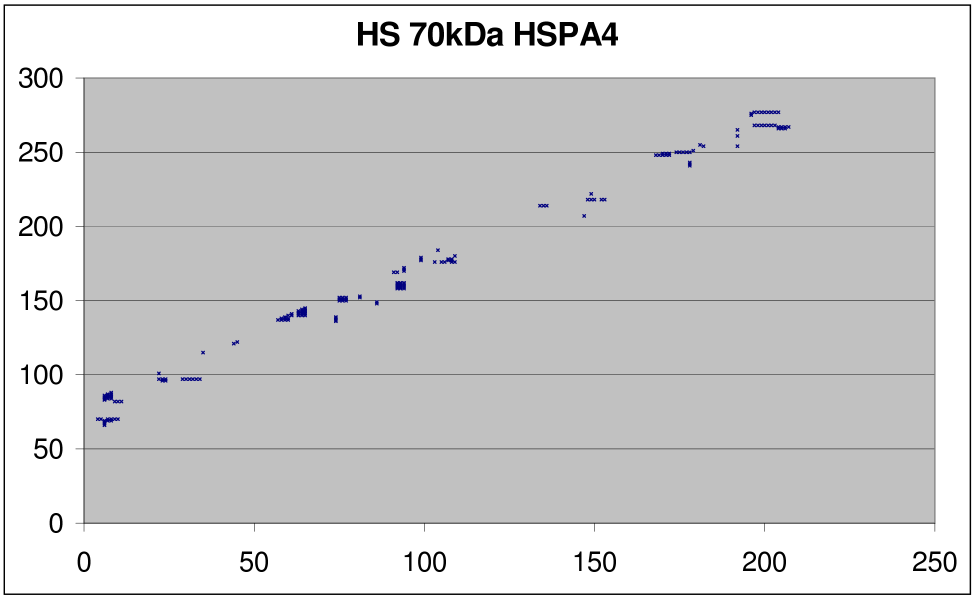

Figure 15: Heat shock protein results for *Homo sapiens*.

In Figure 15 above , structural switching is sporadically distributed over the entire RNA sequence. Groupings of instances of structural change indicative of possible switching behavior occur only around fragments 58 to 65.

In Figure 16, however, a significant cluster of RNA structural activity occurs at fragments 372-384, indicating a suitable candidate for conformational switching.

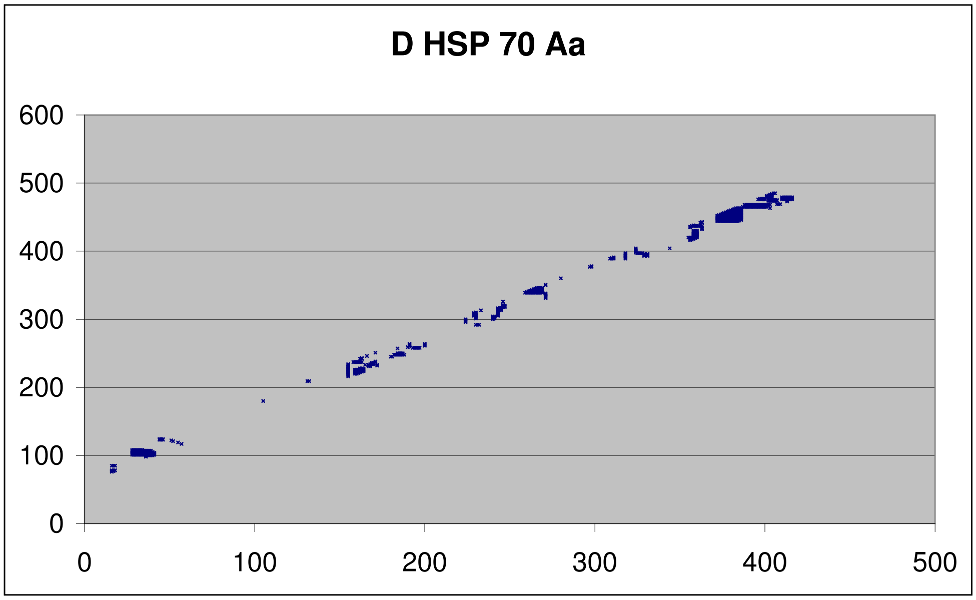

Figure 16: *Drosophila* HSP 70Aa.
